# Nanocrystallization Improves the Solubilization and Cytotoxic Effect of a Poly (ADP-Ribose)-Polymerase-I Inhibitor

**DOI:** 10.3390/polym14224827

**Published:** 2022-11-09

**Authors:** Amer S. Alali, Mohd Abul Kalam, Mohammed Muqtader Ahmed, M. Ali Aboudzadeh, Sulaiman S. Alhudaithi, Md. Khalid Anwer, Farhat Fatima, Muzaffar Iqbal

**Affiliations:** 1 Department of Pharmaceutics, College of Pharmacy, Prince Sattam Bin Abdulaziz University, Al-Kharj 11942, Saudi Arabia; 2 Nanobiotechnogy Unit, Department of Pharmaceutics, College of Pharmacy, King Saud University, Riyadh 11451, Saudi Arabia; 3 Department of Pharmaceutics, College of Pharmacy, King Saud University, Riyadh 11451, Saudi Arabia; 4 CNRS, Institut des Sciences Analytiques et de Physico-Chimie Pour l’Environnement et les Matériaux, University Pau & Pays Adour, E2S UPPA, IPREM, UMR5254, 64000 Pau, France; 5 Department of Pharmaceutical Chemistry, College of Pharmacy, King Saud University, Riyadh 11451, Saudi Arabia; 6 Central Laboratory, College of Pharmacy, King Saud University, Riyadh 11451, Saudi Arabia

**Keywords:** Olaparib, Soluplus, nanocrystals, characterization, stability, MTT-assay, oral-bioavailability

## Abstract

Olaparib (OLA) is an anticancer agent that acts by inhibiting the poly (ADP-ribose)-polymerase-I (PARP-I). Due to its low solubility and low permeability, it has been placed as a BCS Class-IV drug and hence its clinical use is limited. In this study, we develop the nanocrystals of OLA as a way to improve its solubility and other performances. The OLA-NCs were prepared by antisolvent precipitation method through homogenization and probe sonication technique using a novel amphiphilic polymeric stabilizer (Soluplus^®^). Particle characterization resulted approximately 103.13 nm, polydispersity-index was 0.104 with positive zeta-potential of +8.67 mV. The crystal morphology by SEM of OLA-NCs (with and without mannitol) exhibited nano-crystalline prism-like structures as compared to the elongated OLA-pure. The DSC, XRD and FTIR were performed to check the interaction of Soluplus, mannitol and OLA did not exhibit any physical interaction among the OLA, Soluplus^®^ and mannitol that is indicated by the presence of parent wave number peak. Two-fold increased solubility of OLA was found in PBS with Soluplus^®^ from the NCs (69.3 ± 6.2 µgmL^−1^) as compared to pure drug (35.6 ± 7.2 µgmL^−1^). In vitro release of drug from OLA-NCs was higher (78.2%) at 12 h at pH 6.8 and relatively lower (53.1%) at pH 1.2. In vitro cellular cytotoxicity and anticancer effects were examined on MCF-7 cells. OLA-NCs were found effectively potent to MCF-7 cells compared with OLA-pure with approximately less than half IC50 value during MTT assay. Estimation of p53, Caspase-3 and Caspase-9 in MCF-7 cells indicated that OLA-NCs have significantly (*p* < 0.05) increased their expressions. After single oral dose in rats, 12 h plasma drug concentration-time profile indicated approximately 2.06-, 2.29-, 2–25- and 2.62-folds increased Cmax, AUC0-12 h, AUC0-∞ and AUMC0-∞, respectively, from the NCs as compared to OLA-pure. Storage stability indicated that the OLA-NCs was physically and chemically stable at 4 °C, 25 °C and 40 °C up to 6-months. Overall, OLA-NCs were deliberated; its potential feasibility to overwhelm the formulation challenges related to poorly soluble drugs and its future clinical applications.

## 1. Introduction

The Olaparib (OLA) is a poly (ADP-ribose)-polymerase (PARP-I) inhibitor. The PRAP is a key enzyme for the repairing process of DNA-strand breaks. OLA has low solubility and low permeability; therefore, it has been placed as a BCS Class-IV drug. Out of four BCS Classes, due to poor solubility and permeability, the Class-IV drugs are very challenging in achieving their sufficient bioavailability. Among the approaches to improve the solubility and bioavailability of such drugs including the particle size reduction, formulation of the complex of drug-cyclodextrins having the hydrophobic interior and hydrophilic exterior, which could be aqueous soluble, dispersion/adsorption of the drugs onto inert carrier such as mesoporous silica, formulation of such drugs together with high solubilizing agents/surfactants, micronization and nano-sizing or nano-crystallization [1,2,3]. For example, OLA was micronized and prepared as a crystalline solid dispersion in Lauroyl-Macrogol-32 Glycerides and filled in capsules to improve its solubility and bioavailability [4].

A number of drug carriers have been developed to increase the solubility and bioavailability, which in turn enhanced the pharmacological properties of poorly soluble and low permeable drugs. Among these, the nanocrystals (NCs) have fascinated the researcher’s interest to formulate colloidal drug delivery systems with improved pharmacokinetic and pharmacologic properties of the poorly soluble drugs [5].

Nano-crystallization is one of the low cost best techniques to obtain the crystalline particles of low soluble and low permeable drugs in nano-range with simple structures and compositions [3,6]. Due to nano-size range the surface area/volume ratio of NCs are very high, which is the reason for increased solubility and high dissolution rate of any poorly soluble drug in NCs forms [7]. They can be prepared either by top-down method where large sized crystals are reduced to small sized by high pressure homogenization, wet or dry ball mill technique, probe sonication, melt emulsification and solvent evaporation technique or by bottom-up method such as generating nano-sized crystals by precipitation of dissolved drugs [8]. The NCs have been explored for numerous therapeutic uses such as transdermal [9], oral [10,11], ocular [12], systemic such as intravenous administration of asulacrine [13], pulmonary [14], chemotherapy, such as intra-peritoneal injection of paclitaxel [6], and targeted drug delivery systems, such as targeted delivery of camptothecin in MCF-7 xenografted BALB/c mice [14], etc. The suspension of NCs are consisting of crystals of drug in nano-range, stabilizer/surfactant and the dispersion medium such as water, water-ethanol mixture or other non-aqueous media such as polyethylene glycol and oils etc. [10,15].

The NCs-suspension are thermodynamically unstable systems; they tend to flocculate/aggregate/crystal growth to reduce their interaction with water to decrease their free energy. However, these phenomena adversely affect the main characteristics including their nano-size range, high surface area volume ratios and of course the advantages of their manufacturing. Therefore, stabilizers/surfactants were added to decrease the interfacial tension by decreasing the free energy of the nano-systems, thereby prevent the aggregation and flocculation of the NCs by electrostatic or steric stabilization, while they are in suspension form. The plain surfactants such as Poloxamer-188, Tween-80, SLS etc. the polymeric stabilizers such as, polyvinyl alcohol, polyvinyl pyrrolidone, Hydroxy-propyl cellulose, HPMC etc.) or the mixtures of surfactant-polymer can be used for the said purpose [16].

The Soluplus^®^ is a novel polymeric solubilizer having excellent solubilizing property for many BCS-Class-II and Class-IV drugs and produces solid solutions of such poorly soluble drugs [17,18]. It has an amphiphilic chemical structure with average molecular weight (MW) ~90–140 kg·mol^−1^. It is a graft co-polymer of “Polyvinyl Caprolactam-Polyvinyl Acetate: Polyethylene Glycol. Although, it was first synthesized for solubilizing the high concentrations of poorly aqueous soluble drugs (APIs) in amorphous solid dispersions or to design and develop for the solid solutions. Due to sufficiently high HLB value (≈14) of Soluplus^®^, we tried its repurposing as stabilizer for the nano-crystallization of a low soluble low permeable anticancer agent (Olaparib) to improve the overall performance of the developed NCs including the formulation development, in vitro and in vivo bioavailability of the drug. 

Therefore, utilization of Soluplus^®^ as stabilizer for the nano-crystallization of OLA might be considered as the novelty of the present investigation. NCs of OLA can have advantageous properties in terms of its aqueous solubility and/or permeability and hence its bioavailability. Moreover, NCs have ability to prepare solid oral dosage forms.

In this study, we prepared the NCs of OLA by antisolvent precipitation method through homogenization followed by probe sonication techniques, using Soluplus^®^ as stabilizer. The size characterization including the particle/crystal size, polydispersity-index and zeta-potential of the OLA-NCs were performed by differential light scattering, while scanning electronic micrography was done for its morphological characterization. Thermal and diffraction studies were carried out by differential scanning calorimetry and X-ray diffraction studies. The Fourier transform infrared spectroscopy was performed to check alteration (if any) in the functional group of the drug during formulation of NCs. The saturation solubility, drug content, in vitro release of drug, storage stability, in vitro cell line study on MCF-7 cells and in vivo pharmacokinetic studies on rats were performed to check the potential of the developed OLA-NCs.

## 2. Materials and Methods

### 2.1. Materials

The OLA and Tween-80 were purchased from “Mesochem Technology, Beijing, China’’ and “Sigma-Aldrich, St. Louis, MO, USA”. The polymeric stabilizer, “Soluplus^®^ was received as a gift sample from BASF Co., Ltd., Ludwigshafen, Germany”. All other chemicals and solvents were used as received.

### 2.2. Chromatographic Analysis of Olaparib

A reverse phase (RP) high-performance liquid chromatography (HPLC) with UV-detection was used for the quantification of Olaparib (OLA) by adopting the previously reported methods [19,20,21]. Briefly, the HPLC system (Waters^®^ 1500-series controller, Milford, MA, USA) was equipped with a UV-detector (Waters^®^ 2489, dual absorbance detector, USA), a binary pump (Waters^®^ 1525, Milford, MA, USA), automated sampling system (Waters^®^ 2707 Autosampler, USA). The system was monitored by “Breeze software”. A RP, C18 analytical column (Macherey-Nagel 250 mm × 4.6 mm, 5 μm) at 35 °C was used to analyze the drug. The mobile phase was consisted of 50:50 (*v*/*v*) of 0.02 M ammonium acetate solution and methanol, where the pH of the ammonium acetate solution was adjusted to 3.5 by glacial acetic acid. The mobile phase was pumped isocratically at a constant 1.2 mL·min^−1^ flow rate. The injection volume was 30 µL and total run time was 8 min. The standard stock solution of OLA was prepared in methanol (500 μg·mL^−1^) and working standard solutions (1.0–100 μg·mL^−1^) were prepared by serial dilution of the stock solution with the 50:50 (*v*/*v*) mixture of mobile phase. The eluent was detected at 254 nm and the retention time for OLA was at approximately 4.52 min. The regression value of 0.999 indicated that the adopted method was linear accurate and precise over the concentrations 1.0–100 μg·mL^−1^.

### 2.3. Preparation of Olaparib Nanocrystals (NCs)

The previously used antisolvent precipitation-ultrasonication technique was employed to prepare the nanocrystals (NCs) of Olaparib (OLA) in the present study [8,22,23,24]. The drug (25 mg) was dissolved in 200 µL DMSO, the volume was made up-to 2.5 mL by adding 2.3 mL of acetone. The organic solution (2.5 mL) was added drop-wise (at the rate of 1.5 mL·min^−1^) with continuous magnetic stirring (800 rpm) in to 10 mL of aqueous phase containing 25 mg (0.25%) and 50 mg (0.5%) of Soluplus^®^ (a polymeric stabilizer). The stabilizer was dissolved in phosphate buffer saline (PBS, pH 7.4). After getting the homogenous suspension, the organic phase (acetone) was evaporated by continuous magnetic stirring (12 h) at room temperature to get the aqueous suspension. The suspension was centrifuged at 13,500 rpm at 4 °C for 15 min. The supernatant was discarded and the precipitate was further redistributed in an equal volume (10 mL) of PBS. The suspension was homogenized (IKA^®^ WERKE GmBH & Co. KG, Staufen, Germany) for 5 min at 21,000 rpm, followed by probe sonication (Sonics & Materials, Inc., Newtown, CT, USA) for 5 min at 40% power on ice bath to form a homogeneous suspension of OLA in the nano-range. It was further purified by washing (triplicate) with Milli-Q^®^ water to remove the excess stabilizer and collected using ultracentrifugation (Preparative ultracentrifuge, WX-series by Hitachi Koki, Japan) at 4 °C for 20 min and at 30,000 rpm. The obtained pellets of NCs was then resuspended in 10 mL of Milli-Q water containing 2.5% (*w*/*v*) of Mannitol (as cryoprotectant) and freeze-dried (FreeZone-4.5 Freeze Dry System, Labconco Corporation, Kansas City, MO, USA). The freeze-dried (free flowing) NCs was then stored at −80 °C for further characterizations. The aqueous suspension of OLA (OLA-AqS) was also prepared by suspending 10 mg of OLA in Tween-80 (0.25%, *w*/*v*) in 10 mL of PBS (pH 7.4) at 70 °C.

### 2.4. Particle Characterization

The particle-size and polydispersity-index (PDI) of the NCs and OLA-AqS were measured using DLS technique by Zetasizer nanoseries, (Nano-ZS, Malvern Instruments, Worcestershire, UK). The NCs suspension was diluted with Milli-Q^®^ water to get an appropriate concentration and the mean values of particle-size and PDI were obtained after three measurements. Additionally, the zeta-potential (ZP) of the NCs suspension was measured by the same instrument at ambient temperature in the original dispersion medium of the NCs (aqueous solution of stabilizer with and without mannitol). A software (DTS Version 4.1, Malvern, Worcestershire, UK) installed in the instrument automatically measured the electrophoretic mobility of the NCs and transformed it to ZP using the “Helmholtz-Smoluchowski equation”.

### 2.5. Scanning Electron Microscopy (SEM)

The morphological characterization of pure OLA, the OLA-NCs-2 (with and without mannitol) was performed by scanning electron microscopy (SEM) (Zeiss EVO LS10, Cambridge, UK) by following the gold-sputter technique. Here, the freeze-dried NCs were coated with gold in the “Q-150R Sputter Unit” from “Quorum Technologies Ltd. (Laughton, East Sussex, UK)” in Argon atmosphere for 60 s at 20 mA. The imaging was performed at 10–50 KX magnification and 25 kV of accelerating voltage. 

### 2.6. Differential Scanning Calorimetry (DSC)

The differential scanning calorimetry was performed on the pure OLA, Soluplus^®^, mannitol and lyophilized OLA-NCs-2 (with and without mannitol) by DSC-8000 (Perkin Elmer Instruments, Shelton, CT, USA) equipped with software Pyris V-11. Approximately 2–5 mg of weighed samples were hermetically sealed in aluminum pans. The DSC was calibrated with pure Indium (melting point 156.60 °C and heat of fusion 6.80 Cal/g). The DSC thermograms were obtained at the scanning rate of 10 °C·min^−1^ over the temperature range 40–280 °C under an inert atmosphere of N2 (flow rate was 20 mL·min^−1^).

### 2.7. ATR-FTIR Spectroscopy

The ATR-FTIR spectra of pure OLA, Soluplus^®^, mannitol and lyophilized OLA-NCs-2 (with and without mannitol) using the “BRUKER Optik GmBH (Model ALPHA, Bremen, Germany) equipped with OPUS software Version-7.8”. The samples were triturated by mortar and pestle with Potassium bromide (KBr) in 1:100 (*w*/*w*) ratio and compressed (by hydraulic press) into pellets. The FTIR spectra of pellets were acquired from 4000–400 cm^−1^ wavenumber at a resolution of 2 cm^−1^.

### 2.8. X-ray Diffraction Study

To examine the crystallinity of the pure OLA, Soluplus^®^, mannitol and lyophilized OLA-NCs-2 (with and without mannitol), the powdered X-ray diffraction study was done using Ultima-IV Diffractometer (Rigaku, Inc., Tokyo, Japan) over 3° to 60° of 2-theta (2θ) range at the scan rate of 0.5 °·min^−1^. Anode material (X-ray tube) was copper (Cu) with Ka2 elimination of 0.154 nm, monochromatized with a graphite crystal. The diffraction pattern was obtained at the tube voltage of 40 kV and current of 40 mA for the generator with step scan mode (step size 0.02° and counting time was 1 s/step) [25,26].

### 2.9. Solubility of Pure OLA and Its NCs

The saturation solubility of OLA and its NCs was checked by preparing their saturated solution in phosphate buffer (PBS, pH 7.4) and PBS with Soluplus^®^ (0.25% and 0.5%). The excess amount of pure OLA and NCs in triplicate were added into 1 mL of each solvent in glass vials. The mixtures were vortexed for 30 s and put into an orbital shaking (at 100 stroke per min) water bath (Julabo^®^, Allentown, PA, USA) maintained at 37 ± 1 °C for 72 h. Thereafter, vials were taken out and kept at room temperature for 24 h to settle down the undissolved drugs [27]. Then, the supernatants were collected, filtered by 0.45 µ filtration unit (Millipore, Molsheim, France), diluted (if needed) and 30 µL was injected into HPLC-UV system for the quantification of the dissolved drugs.

### 2.10. In Vitro Release Study

The fate of bioavailability of drugs from formulations ultimately depends up on the release of the drug from the formulation to get absorbed into systemic circulation. Therefore, the in vitro release pattern of the drug from OLA-NCs-2 and OLA-AqS were evaluated. Accurately weighed amount of freeze-dried OLA-NCs was suspended into 1 mL of PBS to get 0.1% (*w*/*v*) drug concentration. The release profile of OLA from NCs was compared with the release profile of the drug from it AqS. Each formulation (1 mL) was put into dialysis bags (Spectra/Por^®^ Standard RC Tubing, MWCO 12 KDa). Both end of the bags was closed by closures (Spectra/Por^®^). The dialysis bags were put into beakers (in triplicate) containing 50 mL of release media (pH 1.2 and pH 6.8 with 0.5% *w*/*v*, Soluplus^®^). The beakers were put into shaking (100 stroke/min) water-bath maintained at 37 ± 1 °C. The diffusion of OLA through dialysis bags and its dissolution into release medium was assessed by collecting 1 mL samples from each beaker at set time points. To maintain the sink condition and volume, 1 mL of fresh release medium kept at same 37 °C was replaced in the beakers after each sampling. The collected samples were centrifuged (13,500 rpm for 5 min at 10 °C) and 30 µL of the supernatants were injected into HPLC-UV system to analyze the drug. The percent drug released (%*DR*) was calculated by Equation (1) and the cumulative amount of released drug (%) was plotted against time (h).

(1) $$\%DR=\frac{Conc.\;\left(µg/\mathrm{mL}\right)\times DF\times Volume\;of\;release\;medium\;\left(\mathrm{mL}\right)}{Iniial\;amount\;of\;OLA\;used\;\left(\mu g\right)}\times 100$$

The closeness or differences between the drug release profiles from the two formulations were compared by a “model independent mathematical approach” using “Difference factor (*f*_1_) and Similarity factor (*f*_2_) [28]. The factor “*f*_1_ is directly proportional to the average difference between the release profiles” and “*f*_2_ is inversely proportional to the average squared difference of the release profiles” of OLA from the two formulations [29]. The value of the factors (*f*_1_ and *f*_2_) was calculated by Equations (2) and (3).

(2) $$f_{1}=\frac{\sum_{t\;=\;1}^{n}=Wt\;\left[R_{t}-T_{t}\right]}{\sum_{t\;=\;1}^{n}=\;Wt\;R_{t}}\times 100$$

(3) f2=50×log1+1n  ∑t=1nWt Rt−Tt2−2 1×100

where “*n*” is the total number of samplings or time points, *R_t_* and *T_t_* are cumulative amount of dissolved drug (%) from the reference product (OLA-AqS) and the test product (NCs), respectively, at time “*t*”. The weight factor (*W_t_*) can be adjusted to high or low weights to the selected time-points as required. The *W_t_* was adjusted to 1, because each time-point was weighted equally in the preset investigation [30]. A *f*_1_ value lower than 15.0 and *f*_2_ value of 50.0 or higher than 50.0–100.0, represent the equivalence of the two release curves and thus the performance of the two formulations [31,32].

Moreover, the release data were fitted into some kinetic models (Zero-order, First-order, Korsmeyer–Peppas and Hixson–Crowell Cube Root). The best-fit model for the drug release was chosen based on the highest value of coefficient of correlation (*R*^2^). From the slope and *R*^2^ values of the plots, the value of release-exponent was calculated, which suggest the mechanism of drug release [33,34,35].

### 2.11. Stability Study

The stability study was performed by following the published reports about the nanocrystals to evaluate the stability of NCs in terms of size, PDI, ZP and drug contents [16,36]. Briefly, 10 mg of freeze-dried NCs was packed in amber colored glass containers and stored at 4 °C, 25 °C and 37 °C for 6-months. The changes in the size, PDI, ZP and drug content was evaluated at stipulated times (i.e., at 7th day, 1, 3 and 6 months). The stored NCs was resuspended in PBS (pH 7.4) for evaluating these parameters.

### 2.12. In Vitro Cytotoxicity Studies

#### 2.12.1. Maintenance and Growth of Cells

The MCF-7 cells were procured from the American Type Cutler Collection (Manassas, VA, USA). Cells were grown in 50 cm^2^ tissue culture flasks using “Dulbecco’s Modified Eagle Medium (DMEM) (UFC Biotech, Riyadh, Saudi Arabia)” at 37 °C in an incubator humidified by 5% CO_2_. The media was accompanied with 10% fetal bovine serum (Alpha Chemika, Mumbai, India), 1% mixture of Penicillin (100 units·mL^−1^): Streptomycin (100 µg·mL^−1^) by ThermoFischer Scientific, Waltham, MA, USA, Amphotericin-B (250 ngmL^−1^) by Gibco^®^ (Grand Island, NY, USA) and 1% of L-glutamine (BioWest, Riverside, MO, USA). The MTT was purchased from Sigma Aldrich, St. Louis, MO, USA. The 96-well cell culture plates were to seed the cells with DMEM. 

#### 2.12.2. MTT or Cell Proliferation Assay

Although the OLA (PARP-1 inhibitor) is highly effective to treat the ovarian cancers, which prevented the repair of single-stranded DNA and encourage the conversion of single-stranded-DNA to double-stranded, which causes lethality to the cancer cells [37]. Presently, we do not have the ovarian cancer cells, therefore, the MTT assay was performed on MCF-7 cells to check the potential anticancer efficacy of the OLA-NCs as compared to the pure drug.

The anticancer activity of pure drug (OLA)-AqS and OLA-NCs-2 against MCF-7 were examined by assessing the viability of the treated cells by MTT assay. As the MTT assay estimates the viable cells primarily through the mitochondria-mediated apoptosis. There, this approach was used for determination of anticancer potential of OLA-NCs as compared to pure OLA. The assay was done by observing the enzymatic reduction of a water-soluble dye [3-(4,5-dimethylthiazol-2-yl)-2,5-diphenyltetrazolium bromide] into an insoluble formazan, where the quantitation of formazan designates the relative viability of the cells [38,39]. The cells were seeded in 96 well microplate with 100 μL of medium and allowed to grow overnight. The suspension of OLA and OLA-NCs were prepared, where the concentration of OLA was in the range of 0.75–100 µg·mL^−1^ using the media as diluent [40]. The medium was removed and the cells were exposed for 48 h with the OLA formulations, using a control (composed of the cells with the medium only). After 48 h, the exposed cells with the drug were treated with 100 µL of MTT solution (5 mg·mL^−1^ in PBS). The media from the grown cells were replaced by fresh media without drugs and the cells were further incubated for 4 h at 37 °C after treating with 20 μL of MTT. Thereafter, the culture fluids were thrown away and the crystals of MTT were solubilized in the mixture of DMSO: acetic acid: sodium dodecyl sulfate (99.4 mL: 0.6 mL: 10 g) for 15 min at working temperature. Optical density (OD) was measured at 570 nm using the spectrophotometric microplate reader (Synergy HT, BioTek Instruments, Winooskim, VT, USA). The *cell viability* (%) was calculated by Equation (4). The results were normalized as compared to that obtained for viable control cells (considered as 100%) and represented as the plots of *cell viability* (%) versus log concentration of drug (µgmL^−1^). The IC_50_ values were calculated as Log (inhibitor) versus normalized response on variable slopes through GraphPad Prism V-5.1.

(4) $$Cell\;Viability\;\left(\%\right)=\frac{\left(\mathrm{OD}\;\mathrm{of}\;\mathrm{test}\;\mathrm{samples}\;-\;\mathrm{OD}\;\mathrm{of}\;\mathrm{blank}\right)}{\left(\mathrm{OD}\;\mathrm{of}\;\mathrm{control}-\;\mathrm{OD}\;\mathrm{of}\;\mathrm{blank}\right)}\times 100$$

#### 2.12.3. P^53^, Caspase-3 and Caspase-9 Activities by ELISA

The p53, Caspase-3 and Caspase-9 activities were measured by ELISA kits following the manufacture’s protocol and reported works [41,42]. The MCF-7 cells (5 × 10^4^ cells/well) were seeded in 96-well plates. The seeded cells were treated with the AqS of OLA-pure and OLA-NCs. The concentration of OLA was kept 10 µgmL^−1^, which was approximately near to the IC_50_ value of the OLA-NCs-2 (9.71 µgmL^−1^). The untreated cells were served as control. All the treated cells were allowed to equilibrate at ambient temperature. Approximately 100 µL of p53, Caspase-3 and Caspase-9 reagents were transferred into the respective wells of the plate containing 100 µL of culture media. The plates were covered and stirred for 1–2 min at 500 rpm and then incubated at ambient temperature. After 1 h of incubation the OD was measured at 405 nm wavelength by microplate reader.

### 2.13. Pharmacokinetic Study

Male Wistar rats were used for this study. The Standing Committee of Bioethics Research (SCBR) at Prince Sattam bin Abdulaziz University, Deanship of Scientific Research has granted the animal ethical approval (SCBR-030-2022) for in vivo studies on rats. Twelve rats were divided in two groups. Group-1 for OLA-AqS and Group-II for OLA-NCs. Each animal of the respective groups received OLA-AqS and OLA-NCs individually. Oral route was selected for the administration of all OLA formulations at 25 mg·kg^−1^ of body weight equivalent dose of OLA was given orally to all animals. At predetermined time points (0.25, 0.5, 1, 3, 6 and 9 h) approximately 0.5 mL of blood was aspirated through retro orbital plexus from each animal. The samples were collected in heparinized tubes and centrifuged at 6000 rpm for 10 min and at 4 °C to obtain the plasma. Approximately 200 µL supernatant (plasma) was collected and stored at −80 °C till analysis was performed.

### 2.14. Chromatographic Analysis

Previously reported RP-HPLC method was used for the analysis of OLA in the plasma samples [7,20]. In brief, the HPLC system (Waters^®^ 1500 Series, Milford, MA, USA) was fitted out with UV-dual absorbance detector (Waters^®^ Model-2489), Binary pump (Waters^®^ Moel-1525), Autosampler (Waters^®^ Model-2707 plus). The HPLC-UV system was monitored and controlled by the “Breeze software”. A C18 Waters symmetry column (150 × 4.6 mm, 5 µm, particle size) was used for the elution of OLA and Paclitaxel (used as internal standard, IS) [7]. The mobile phase composed of ammonium acetate buffer (pH was adjusted to 3.5 by Orthophosphoric acid) and Methanol at 50:50 (*v*/*v*) was pumped at isocratic mode at the flow rate of 1 mL·min^−1^. The UV-detection wavelength was 254 nm (for OLA) and 230 nm (for IS). The column heater temperature was set to 35 °C and the volume of injection was 30 µL.

#### Sample Preparation

Protein precipitation method (simple with high recovery) was used for the drug extraction from the collected plasma samples. The calibration curve samples were prepared by pipetting 50 μL of working stock solution of OLA (in methanol) and 20 μL of IS (20 µg/mL) into 140 μL of plasma and the mixture was vortexed for 30 s. Acetonitrile (290 μL) was added into the obtained mixture for protein precipitation, vortexed for 2 min and then centrifuged at 13,500 rpm for 10 min. Approximately 200 µL of the supernatant was collected and 30 µL of the supernatant was injected into HPLC system for the quantification of the drugs in the samples.

### 2.15. Statistical Analysis

The results were represented as mean with standard deviation (± SD), unless otherwise indicated. The statistical analysis was performed using GraphPad Prism: Version-5.1 (GraphPad Software, Inc., San Diego, CA, USA). The pharmacokinetics parameters was calculated by “PK-Solver Software, Nanjing, China using MS-Excel-2013” through the non-compartmental approach. The comparative analysis of the data was accomplished by Student’s *t*-test and *p* < 0.05 was considered as statistically significant, unless otherwise indicated.

## 3. Results and Discussion

### 3.1. Chromatographic Analysis of OLA

A slight modification in the reported HPLC-UV methods [19,20,21] was successful for the quantification of OLA during the in vitro experiments. The calibration curve was prepared between the drug concentrations (μg·mL^−1^) versus the corresponding peak areas during the chromatographic analysis. The calibration curve of OLA was linear in the concentration range of 1.0–100 μg·mL^−1^ with correlation co-efficient or *R*^2^ = 0.9998 and the straight-line equation was Y = 20,052x − 14,368. The regression equation was found worthy for the calculation of OLA concentrations in the samples of in vitro experiments. A mixture of 0.02 M ammonium acetate solution and methanol (50:50, *v*/*v*) at isocratic mode (at 1.2 mL·min^−1^ flow rate) was suitable mobile phase for the assay of OLA.

### 3.2. Formulation and Characterizations of OLA-Nanocrystals

Two batches of OLA-NCs were prepared by keeping the constant amount of drug (25 mg) and varying the amounts of Soluplus^®^ as (i.e., 25 mg and 50 mg, respectively). Among the two formulations, NCs-2 (0.5% *w*/*v*, i.e., 50 mg Soluplus^®^) was having better physical characteristics (Table 1). The size of NCs-2 was smaller (92.43 ± 7.02 nm), uniformly distributed with low PDI value (0.104 ± 0.061) as compared to NCs-1 (0.25% *w*/*v*, i.e., 25 mg Soluplus^®^). The smaller size of NCs-2 might be associated with the higher concentration of Soluplus^®^, which provided better stabilization to the NCs of OLA as compared to NCs-1 where the amount of Soluplus^®^ was 25 mg and the amount of OLA was same (i.e., 25 mg). In addition, the higher concentration of Soluplus increase the stability of NCs-2 by minimizing the super-saturation level and metastable-zone, which is a prime reason for crystal growth and nucleation [43]. This was further justified as it has shown excellent solubilizing properties to several BCS class-II drugs and provided opportunities to produce solid solutions of poorly aqueous soluble drugs [18,44].

**Figure 1:**
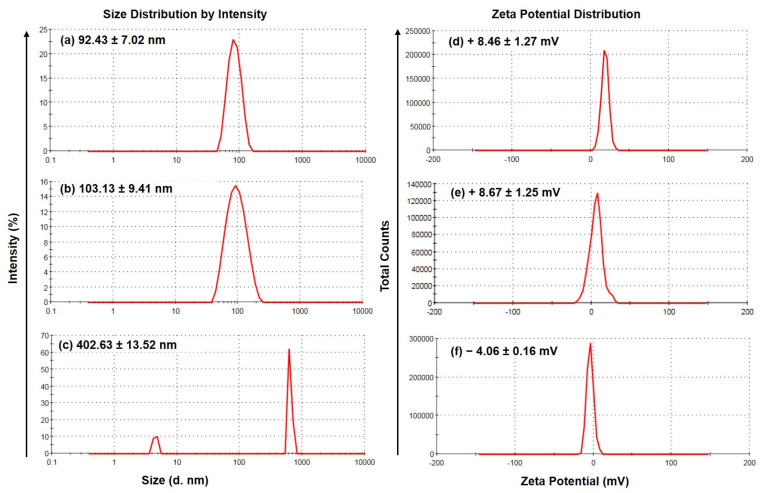
Size and zeta potential distributions of OLA-NCs-2 (**a**,**d**), respectively; OLA-NCs-2 with mannitol (**b**,**e**), respectively, and OLA-aqueous suspension (**c**,**f**), respectively. Data are expressed as mean of three measurements with standard deviation (Mean ± SD, *n* = 3).

The size and zeta potential distributions of the optimal formulation i.e., OLA-NCs-2 (with and without mannitol) as compared to the aqueous suspension of pure drug (OLA-AqS), have been illustrated in Figure 1. The absolute zeta-potentials were almost same in both the cases (+8.46 to +8.59 mV). The crystal size in case of NCs-2 was much smaller (92.43 ± 7.02 nm) (Figure 1a), as compared to the NCs-1 (176.03 ± 8.81 nm) (Figure 1b) and they were uniformly distributed in both the NCs. Relatively, their sizes were much smaller than the crystal size of the drug in the OLA-AqS, which were approximately 402.63 ± 13.52 nm (maximum population) but they have bimodal distribution as shown in Figure 1c. Although the absolute zeta potential values of the NCs were not high enough rather they have slightly positive surface charges (Figure 1d,e) which may interact with the cellular surfaces, while the zeta potential of the OLA-AqS was low and negative (−4.06 mV) (Figure 1f). The positively charged OLA-NCs may interact electrostatically with the cellular layer that would prolong their retention with the cells, which facilitate the enhanced trans-cellular passage and cellular uptake and ultimately would improve the bioavailability of the drug. The absolute ZPs of the NCs were, so it would not provide sufficient colloidal stability to the NCs at nano-suspension condition, hence freeze-dried samples of the OLA-NCs could be stored for better stability and prolong shelf-life. The slight changes (although not significant) in the physical characteristics of OLA-NCs-2 after freeze-drying with 1% mannitol were noted which were summarized in Table 2.

Freeze-drying provide stabilization to the OLA-NCs by avoiding the unstable factors such as Ostwald ripening and aggregation in the aqueous environment. Additionally, the dried form of nano-crystals can be fabricated into other dosage forms such as sterile powder for reconstitution to use as injection/ophthalmic preparations, filled in capsules and can be compressed into oral tablets [45]. On the other hand, the freeze-drying may consequence the solidification of the NCs and stabilizer, which may lead to irreversible clumping of NCs [46]. Hence, mannitol as cryoprotectant was added in to the suspension of NCs prior to freeze-drying the optimal OLA-NCs to prevent the incidence of such unusual occurrence and to get easily re-dispersible OLA-NCs at the time its use [47].

Thus, the OLA-NCs prepared with 0.5%, *w*/*v* of the stabilizer by homogenization (at 21,500 rpm for 10 min) followed by 60 s probe sonication (at 40% power) was found to have better physical characterization. The relatively higher drug content analysis also advised that the NCs-2, was the optimal formulation, as there was a minimum loss of the drug in this case. 

The antisolvent precipitation method (Bottom-up technique) followed by solvent evaporation was adopted for the nano-crystallization of OLA. It is poorly aqueous soluble drug, so at first the drug was dissolved in was dissolved in DMSO (200 µL) and vortexed after adding 2.3 mL of Acetone, served as organic phase. The aqueous phase was consisted of 10 mL of a polymeric stabilizer (Soluplus^®^). Due to the addition of organic phase (solvent) into the aqueous phase (antisolvent) precipitation occurred. The evaporation of organic solvent was performed by overnight continuous magnetic stirring. The continuous magnetic stirring, homogenization and probe sonication could generate the crystals in the nano-range.

During the homogenization and sonication, high turbulence and forceful mixing of the two liquid phases occur. At the same moment the anti-solvent causes the precipitation of the drug (OLA) as fine crystalline structures in the nano-range [48]. In this method the ratios of solvent: anti-solvent and drug: stabilizer, any interaction between drug and stabilizer and the HLB-value of stabilizer/surfactant are the vital process parameters [49]. The stabilizers/surfactants with high HLB-values are considered as the best one for the nano-crystallization of highly lipophilic or poorly aqueous soluble drugs [50]. Therefore, we have chosen Soluplus^®^ for the nano-crystallization of OLA in this study. It is a simple, low cost and low energy consumption method. Due to the simplicity and low heat generation, this method could be adopted for the heat-labile drugs without their unwanted degradation [43].

**Figure 2:**
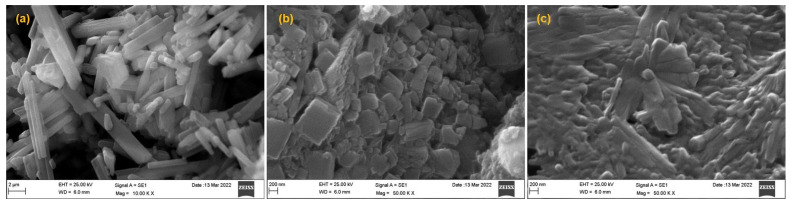
Scanning electron micrographs of pure OLA (**a**); OLA-NCs-2 prepared with 0.5%, *w*/*v* of Soluplus^®^ as stabilizer (**b**) and OLA-NCs-2 freeze-dried with mannitol (**c**).

The Soluplus^®^ at 0.5% concentration generated smaller crystals of OLA in the nano-range (92.43–176.03 nm). Similar findings were also reported in by [51,52]. The shape and size of the pure OLA, OLA-NCs-2 (with and without mannitol) can be seen in the SEM images (Figure 2). The shape and size of pure OLA was elongated and larger crystalline (Figure 1a), where the size and shape were further decreased after the process of nano-crystallization with the stabilizer (Figure 1b) and the morphology of freeze-dried OLA-NCs-2 with mannitol was little hazy (Figure 1c), which might be due the covering of the NCs by the cryoprotectant during freeze-drying. The results of scanning electron micrographs were almost in agreement with the crystal size characterization of the OLA-NCs by Zetasizer, indicating that the process parameters and formulation variables for the nano-crystallization were properly optimized.

### 3.3. DSC Analysis

**Figure 3:**
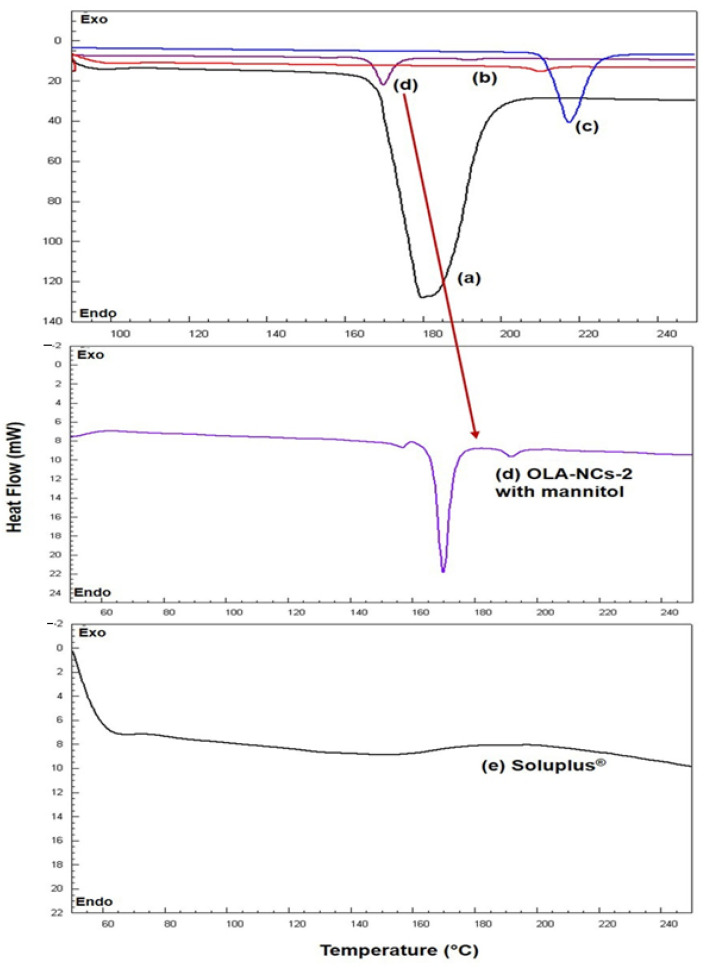
DSC endotherms of mannitol (black color) (**a**); OLA-NCs-2 (red color) (**b**); pure OLA (blue color) (**c**); OLA-NCs-2 with mannitol (dark red color) (**d**) and Soluplus^®^ (grey color) (**e**).

The DSC thermograms for pure OLA, Soluplus^®^, mannitol, PM and NCs-2 were represented in Figure 3. The thermogram of mannitol (black color) indicated a sharp endothermic peak at 177.72 °C [53]. The DSC scan of pure OLA has shown a sharp endothermic peak at 217.19 °C (blue color) corresponding to its melting temperature, which was expected for an anhydrous crystalline substance, and almost similar to its reported melting point (199–206 °C). The scan of Soluplus (grey color) did not show any specific endothermic peak throughout the temperature range (50–250 °C). The thermogram of OLA-NCs-2 (red color) has shown a less intense endothermic peak at approximately 209.52 °C. A slight shifting of the endothermic peak of pure OLA towards left was noted in the thermogram of OLA-NCs with less intensity almost near to its melting temperature indicating the less-crystalline nature of OLA in the NCs form.

In the DSC scan of OLA-NCs-2 with mannitol (dark red color) a slightly less intense endothermic peak of OLA was appeared with slight shifting towards lower melting temperature (190.77 °C) as compared to the melting temperature of pure drug (217.19 °C) in association with a sharp endothermic peak of mannitol at 177 °C. A slight shifting towards lower melting temperature of OLA in case of OLA-NCs-2 (with and without mannitol) indicated that the crystallinity of OLA was not much affected during the process of nano-crystallization. The decreased melting temperature of OLA in NCs was due the reduced crystal sizes and decreased crystal lattice energy of OLA after nano-crystallization. The reduced lattice energy might be the due to the interaction of OLA molecules in the lipophilic micellar zone due to sufficiently high HLB stabilizer which was also found when the atorvastatin was nano-crystallized by a non-ionic surfactant (Pluronic F-68) as stabilizer [52]. The appearance of endothermic peaks of OLA almost near to its melting temperature in NCs-2 and NCs-2 freeze-dried with mannitol, indicated that the there was no potential interaction occurred between the drug and the used excipients. Overall, the DSC results confirm the reduced crystallinity of OLA in NCs state, which must improve the aqueous solubility of the drug, which in turn increase the drug absorption and bioavailability in accordance to previously reported similar studies [54,55].

### 3.4. FTIR Analysis

**Figure 4:**
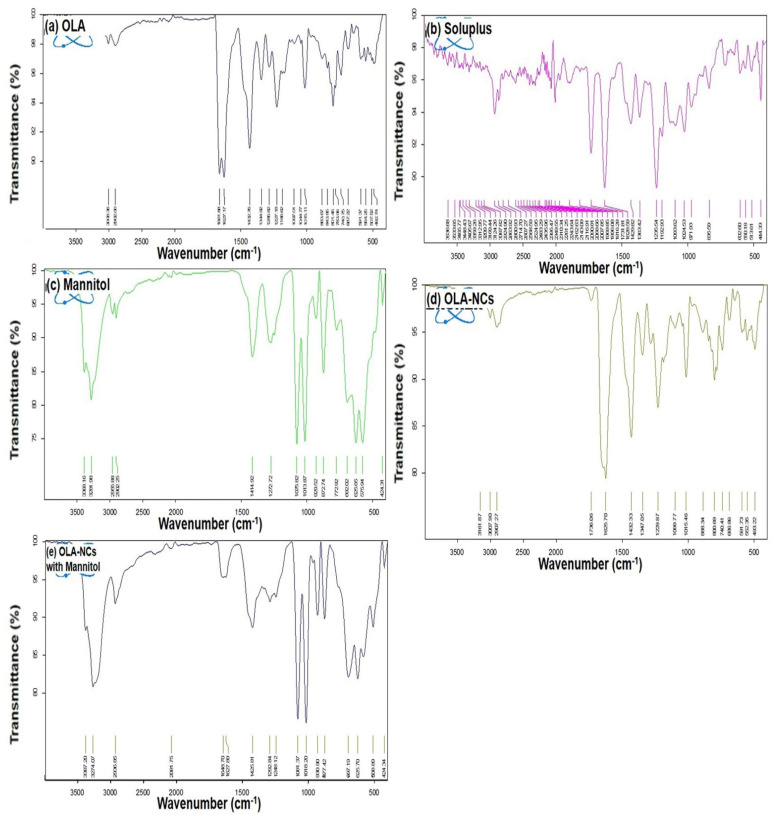
FTIR-spectra of pure OLA (**a**); Soluplus^®^ (**b**); mannitol (**c**); lyophilized OLA-NCs-2 (**d**) and lyophilized OLA-NCs-2 with mannitol (**e**).

The FTIR spectrum of OLA pure (Figure 4a) exhibiting the different absorption peaks at 3513, 3163, 1681, 1651, 1438, 1222, 1012, 810, 773, 587 cm^−1^, and at 1625 and 1725 cm^−1^ for Soluplus^®^ (Figure 4b). The FTIR spectrum of mannitol (Figure 4c) has characteristic absorption bands at 1413.02, 1075.08 and 1011.63 cm^−1^. The spectrum of OLA-NCs (Figure 4d) indicated absorption bands at 3515, 1690, 1681, 1675, 1222 and 773 cm^−1^. The spectrum of our formulation (OLA-NCs freeze-dried with mannitol) exhibited the presence of cyclopropane carbonyl ring attached with piperazine, which is an indicative of aromatic primary amine, NH stretching 3515 cm^−1^ as shown in Figure 4e. The presence of quinone or conjugated ketones with wavenumbers 1690, 1675 and 1681 cm^−1^. The wavenumber 1651 cm^−1^ was due to the presence of pthalazone. The wavenumber 1222 cm^−1^ for 4-Fluorobenzyl group. The wavenumbers 810 cm^−1^ and 773 cm^−1^ were due to the cyclopropane nucleus. The presence of cyclopropane carbonyl ring attached with piperazine, quinone, pthalazone, 4-Fluorobenzyl group and cyclopropane nucleus wave numbers indicated the presence of OLA in OLA-NCs.

The presence of traces of Soluplus^®^ in OLA-NCs exhibited the regions of carbonyl (CO) stretching at carbonyl ester and carbonyl amide group at 1625 cm^−1^ and 1725 cm^−1^, respectively, for caprolactam ring in Soluplus^®^. We have observed the distinguished CH stretching at 1413.02 cm^−1^ and C-O stretching at 1011.63 cm^−1^ as well as at 1075.08 cm^−1^, which were shown the presence of mannitol in freeze dried OLA-NCs. The results were similar to previous reports where OLA, Soluplus^®^ and mannitol have shown similar spectra of different functional groups exhibiting no interaction among the drug, stabilizer and cryoprotectant, which was evidenced by the presence of their parent absorption bands at around the same wavenumber [56,57,58,59]. These results also confirm that the nano-crystallization of OLA could not alter the basic molecular structure of the drug during the process of homogenization.

### 3.5. XRD Analysis

**Figure 5:**
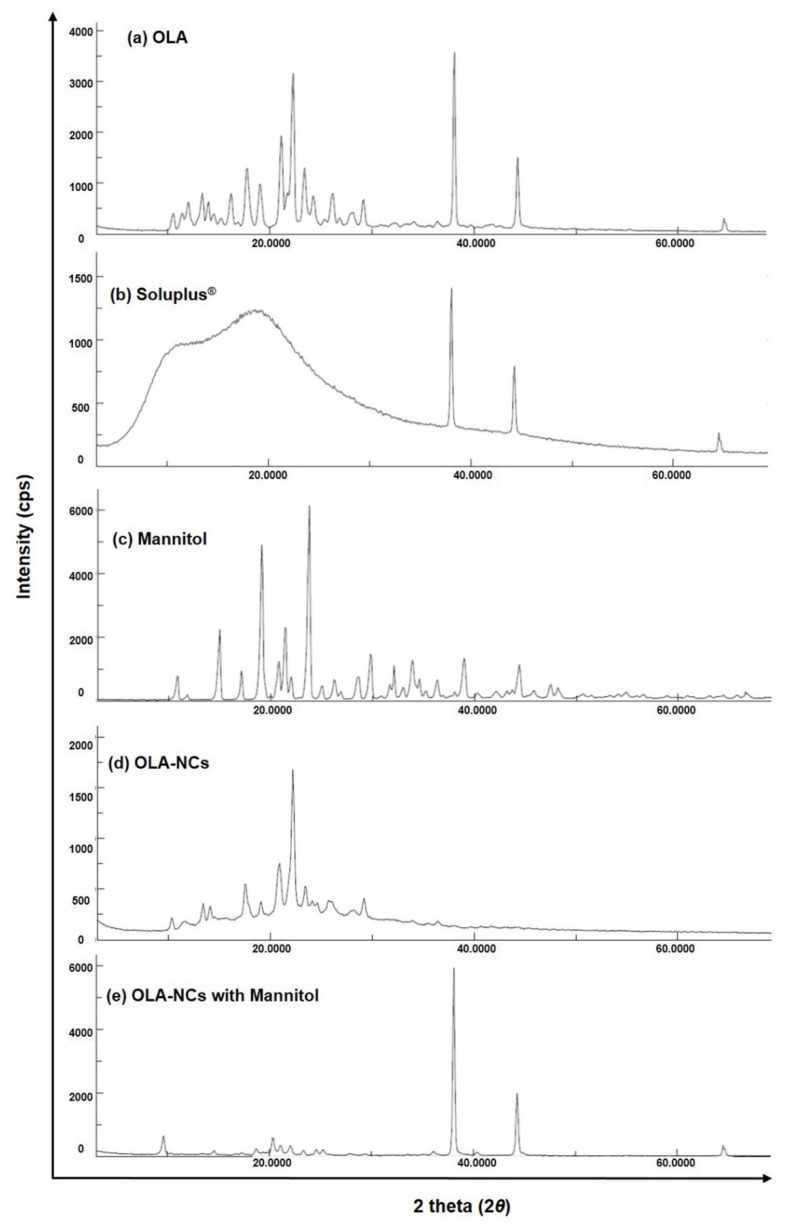
XRD diffractograms of pure OLA (**a**); Soluplus^®^ (**b**); mannitol (**c**); lyophilized OLA-NCs-2 (**d**) and lyophilized OLA-NCs-2 with mannitol (**e**).

The X-ray diffractograms of pure OLA, Soluplus^®^, mannitol, lyophilized OLA-NCs-2 and lyophilized OLA-NCs-2 with mannitol are illustrated in Figure 5. The diffractogram of pure OLA (Figure 5a) has shown characteristic intense diffraction peaks at 2θ of 17.7°, 21.1°, 22.3°, 38.8° and 44.2° with Bragg’s spacing (or d-values) of 5.0068, 4.207, 3.9833, 2.366 and 2.0474 and the intensities of 679 cps (I/I_0_ 27), 1061 cps (I/I_0_ 42), 1780 cps (I/I_0_ 70), 2570 cps (I/I_0_ 100) and 1004 cps (I/I_0_ 40) demonstrating the crystal structure of OLA [37]. The X-ray diffractogram of Soluplus^®^ (Figure 5b) has characteristic diffraction peaks at 2θ of 38.0°, 44.2° and 64.5° with d-values of 2.366, 2.0474 and 1.4435 and the intensities of 819 cps (I/I_0_ 100), 400 cps (I/I_0_ 49) and 142 cps (I/I_0_ 18) advising the low crystalline character of the amphiphilic stabilizer. The diffractogram of mannitol (Figure 5c), showing the diffraction peaks at 2θ of 15.0°, 19.1°, 21.4°, 23.8°, 29.8°, 39.0° and 44.4° with d-values of 5.901, 4.6428, 4.1487, 3.7355, 2.9957, 2.3076 and 2.0326 and the intensities of 1814 cps (I/I_0_ 36), 4060 cps (I/I_0_ 80), 1861 cps (I/I_0_ 37), 5092 cps (I/I_0_ 100), 1177 cps (I/I_0_ 24), 1037 cps (I/I_0_ 21) and 868 cps (I/I_0_ 18) indicating the crystallinity of pure mannitol. The XRD-pattern of OLA-NCs-2 in Figure 5d, showing the less intense diffraction peaks at 2θ of 17.5°, 20.9°, 22.0° and 22.2° with d-values of 5.0635, 4.2468, 4.0369 and 4.001, and with the intensities of 213 cps (I/I_0_ 25), 280 cps (I/I_0_ 33), 338 cps (I/I_0_ 39) and 872 cps (I/I_0_ 100). The diffraction peaks at for NCs were almost appeared at the same 2θ values (± 0.3) as seen in the diffractogram of pure OLA with little low intensities, indicating that the crystallinity of the drug was remained (with low intensities) in the nanocrystal form of the drug. The diffractogram of OLA-NCs-2 lyophilized with mannitol (Figure 5e), showing the peaks at 2θ of 9.6°, 20.3°, 38.1°, 44.3° and 64.5° with d-values of 9.2053, 4.371, 2.36, 2.043 and 1.4435 with the intensities of 332 cps (I/I_0_ 9), 318 cps (I/I_0_ 8), 4080 cps (I/I_0_ 100), 1406 cps (I/I_0_ 35) and 294 cps (I/I_0_ 8). The appearance of mixed peaks of OLA and mannitol in this case suggesting that the, there was no any adverse interaction occurred between the drug and the cryoprotectant.

Overall, the existence of characteristic intense peaks of OLA in the XRD-pattern of freeze-dried OLA-NCs-2 with and without mannitol, proposing that the process parameters did not affect the crystalline character of the drug, which was also reported during the co-crystallization of Olaparib and other PARP-inhibitors [40,60]. Around the same 2θ values the decreased intensity peaks were suggestive of the reduced crystallinity of OLA in the NCs, which might be attributed to the surface covering of the drug crystals by the stabilizer. The XRD studies endorsed the reduced crystallinity of OLA in NCs, which was further confirmed by the improved saturation solubility of the OLA-NCs in PBS with Tween-80 and PBS with Soluplus^®^ as compared to pure drug.

### 3.6. Solubility Determination

**Figure 6:**
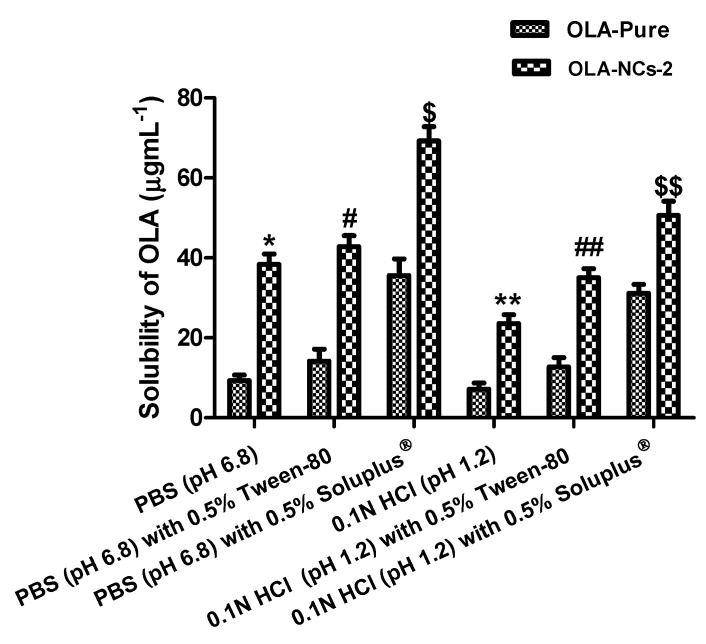
Solubility of Olaparib (OLA) in different solvents. All the data are expressed as mean of three measurements with standard deviation (Mean ± SD, *n* = 3) * *p* < 0.05 OLA-NCs-2 vs. OLA-pure in PBS; ^#^ *p* < 0.05 OLA-NCs-2 vs. OLA-pure in PBS with 0.5% Tween-80 and ^$^ *p* < 0.05 OLA-NCs-2 vs. OLA-pure in PBS with 0.5% Soluplus^®^. ** *p* < 0.05 OLA-NCs-2 vs. OLA-pure in 0.1N HCl (pH 1.2); ^##^ *p* < 0.05 OLA-NCs-2 vs. OLA-pure in 0.1N HCl (pH 1.2) with 0.5% Tween-80 and ^$$^ *p* < 0.05 OLA-NCs-2 vs. OLA-pure in 0.1N HCl (pH 1.2) with 0.5% Soluplus^®^.

The saturation solubility of pure OLA was 9.3 ± 2.4 µg·mL^−1^, 14.2 ± 5.1 µg·mL^−1^ and 35.6 ± 7.2 µg·mL^−1^ in PBS, PBS with Tween-80 and PBS with Soluplus^®^, respectively. The significant (*p* < 0.05) increase in the solubility of OLA was noted from the nanocrystals (OLA-NCs-2), which were 38.3 ± 4.6 µg·mL^−1^, 42.8 ± 4.5 µg·mL^−1^ and 69.3 ± 6.2 µg·mL^−1^ in PBS, PBS with Tween-80 and PBS with Soluplus^®^, respectively. The solubility of OLA was 7.1 ± 2.7 µg·mL^−1^, 12.7 ± 3.9 µg·mL^−1^ and 31.1 ± 3.9 µg·mL^−1^ in 0.1N HCl (pH 1.2), 0.1N HCl with Tween-80 and 0.1N HCl with Soluplus^®^, respectively. An increased solubility of the drug was found from the nanocrystals (OLA-NCs), which were 23.5 ± 3.8 µgmL^−1^, 35.1 ± 3.9 µg·mL^−1^ and 50.6 ± 6.2 µg·mL^−1^ in HCl, 0.1N HCl with Tween-80 and 0.1N HCl with Soluplus^®^, respectively. The solubility of OLA in different solvents was well represented in Figure 6. The increased solubility of OLA from NCs was due to the nano-sizing of the drug. The small sized particles provided an overall increased surface area which come in contact with the solvents. Thus, the contact points for the drug-NCs with the solvents were increased, which supported the dispersibility and wetting of drug-NCs [61] and hence amplified the drug solubilization from its NCs form.

In addition, the improved solubility of OLA from NCs was because of the strong affinity between the drug and the stabilizer (Soluplus^®^) to generate the “molecular dispersion” which was responsible for modifying the saturation solubility and solubility equilibrium of the drug. This outcome show a relationship with “Noyes Whitney Equation” that describes the dependence of relative solubility of particles (NCs) with variable radii and the concentration of the dissolved solutes [62]. A significant (*p* < 0.05) increase in the solubility of OLA in PBS with the surfactants (Tween-80 and Soluplus^®^) would support an increased in vitro release of the drug using the same solvent as release media. Overall, the solubility of OLA was higher at pH 6.8 than at pH 1.2, because it has pH dependent solubility which is higher at pH 6.8 that simulates the intestinal fluid (pH 6.5–7.8). Since at low concentrations, OLA has the propensity for the P-glycoprotein (Pgp)-mediated efflux but at high concentrations (>260 μM), the efflux is saturated. As the solubility of OLA at intestinal fluid pH is relatively higher (0.16 mg·mL^−1^), therefore the Pgp-mediated efflux would be saturated and drug absorption would not be influenced [4,60,63]. Conclusively, the enhanced saturation solubility would facilitate the overall absorption and bioavailability of OLA.

Where, OLA = Olaparib; NCs = Nanocrystals; HCl = Hydrochloric acid and PBS = Phosphate buffer saline.

### 3.7. In Vitro Drug Release

**Figure 7:**
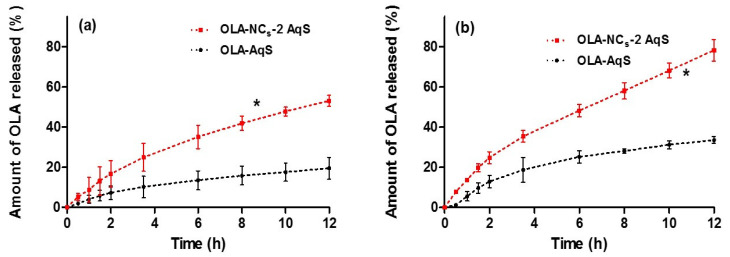
In vitro release of OLA from the aqueous suspensions of lyophilized OLA-NCs-2 and pure OLA in 0.1 N HCl acid (pH 1.2) with 0.5% Soluplus^®^ (**a**) and in PBS (pH 6.8) with 0.5% Soluplus^®^ (**b**). Data are represented as mean of three readings with standard deviation (Mean ± SD, *n* = 3) and “*” *p* < 0.05; OLA-NCs-2 was significant as compared to pure OLA-AqS.

The in vitro drug release study deliberates the practical ability of the probable in vivo performance of dosage forms. Due to poor aqueous solubility of OLA, this study was performed in biological buffers with Soluplus^®^, where Soluplus^®^ was added in the buffers (0.1 N HCl, pH 1.2 and PBS, pH 6.8) to enhance the dissolution of the drug. The release profiles of the OLA-formulations were presented in Figure 7. The release profiles indicated a significantly (*p* < 0.05) increased dissolution of OLA from NCs as compared to the pure OLA-AqS (Figure 7a,b). A comparatively higher release was observed at pH 6.8 (Figure 7b) than that occurred at pH 1.2 (Figure 7a). The difference in the release profiles at two different pH was due to the fact that OLA has pH dependent solubility. At pH 6.8 it (whether NCs or pure-OLA) has higher solubility as compared to pH 1.2, which was also observed in the comparative release profiling of the co-crystals of OLA with Fumaric acid and pure-OLA [40,60].

At pH 1.2, approximately 19.5% of drug was released at 24 h from pure OLA-AqS, while almost an equal amount of drug (19.0%) was released from the OLA-NCs within 2 h and up to 24 h approximately 53.6% of the drug was released from OLA-NCs (Figure 7a). At pH 6.8, approximately 35.8% of OLA was released at 24 h from pure OLA-AqS, while an equivalent amount of drug (35.5%) was released from the OLA-NCs within 3.5 h and up to 24 h approximately 86.5% of the drug was released from the NCs (Figure 7b).

From the saturation solubility study, we found that the OLA exhibited a typical pH-dependent solubility which was relatively higher at pH 6.8 (38.3–69.3 μg·mL^−1^) and lower at pH 1.2 (23.5–50.6 μg·mL^−1^). The release profile of OLA was consistent and in agreement with the solubility profiles, where release of drug from NCs was higher (86.4 ± 5.7%) till 24 h at pH 6.8 (simulating the intestinal fluid pH) and relatively lower (53.6 ± 2.3%) during the same duration at pH 1.2 (simulating the gastric fluid pH). The reason for higher release of OLA at pH 6.8 might be the abundance of buffer species of the release media (PBS) which influenced the solubility of the drug and subsequently the percentage of drug released by making OLA salts of high solubility. Furthermore, the solubility and release of OLA was increased due to the increased ionic concentrations while at low buffer capacity (pH 1.2) a decrease in the solubility and release rate was observed [64].

The increased rate of drug release from NCs was the consequences of the nano-sizing of OLA (92.43 nm to 103.13 nm) which provided an enhanced contact of the overall enlarged surface area of the NCs with the release medium, provided higher saturation solubility to the drug which in turn could give its high dissolution [16]. In addition, the stable and nano-size range of NCs stopped aggregation of the crystals and expedited their surface wettability due to the presence of solubilizer (Soluplus^®^) and hence the dispersibility and dissolution to OLA [52]. The presence of Soluplus^®^ at the surfaces of OLA-NCs might reduce the interfacial tension between drug and the amphiphilic polymeric solubilizer/stabilizer through hydrogen bonding with the water molecules [65].

Apart of these, higher and prolonged release of OLA-NCs can be endorsed because of the multi-molecular micellar formation of the amphiphilic Soluplus^®^ (due to high HLB) as observed during the in vitro release of poorly soluble atorvastatin-NCs in presence of Poloxamer-188 which is also a high HLB surfactant [52]. The hydrophobic part of the micelles of Soluplus^®^ could interact with the OLA through the “Van-der Waals intermolecular forces” and decrease the fast diffusion of the drug from inside of the core of micellar assemblies [66].

The values of difference (*f*_1_) and similarity factor (*f*_2_) for the release of OLA-NCs at acidic release media (pH 1.2) were 158.9 (i.e., >15) and 34.9 (i.e., <50), respectively, while these were 110.8 (>15) and 30.7 (<50) at pH 6.8. The *f*_1_ and *f*_2_ values suggested that OLA-NCs had a completely different release pattern than that of the AqS of pure OLA in the same release medium. Similar findings were also observed during the release study of Cyclosporin-A micelles [67], spherical crystals of poorly soluble fenbufen [68], multiple brands of amoxicillin [32] and aminophylline [69] in dissimilar release media.

The release pattern of OLA from NCs form was investigated by applying the release kinetics equations. Applying the kinetic models, it was found that the release of OLA from NCs could be explained well by two models (Korsmeyer–Peppas followed by first-order kinetics). The curve between log time versus log fraction of drug released (Korsmeyer–Peppas) and log drug remaining (%) vs. time (First order) were found to be linear as shown in Figure S1. The linearity in the release profiles (as suggested by the above two release models) indicated the prolonged release of the drug from NCs. Amongst the applied release models, the highest value of coefficient of correlations (*R*^2^ = 0.9541 and 0.9764, with Korsmeyer–Peppas model, while 0.8035 and 0.9592 with first order at pH 1.2 and pH 6.8, respectively) as shown in Table 3. In view of the values of slopes *R*^2^ obtained for different models, the release-exponents were also calculated and the obtained release-exponents suggesting that the mechanism of drug release from NCs was Fickian–Diffusion type [33,34,35]. All the values of coefficient of correlations and release-exponents are summarized in Table 3.

### 3.8. Stability Studies

The NCs-2 was evaluated at different time intervals for its physical and chemical stability to ensure the limits of stability, stored at 4 ± 2 °C, 25 ± 1 °C and 40 ± 1 °C for a period of 6 months, results are presented in Table 4. The results of physical stability did not show significant changes in the size, PDI, zeta-potential and OLA content at 4 ± 2 °C, but a slight increase in the size was noted at 25 ± 1 °C and 40 ± 1 °C at 3 and 6 months. The crystal growth of NCs-2 might be a reason for increased sized at slightly higher temperature even in the lyophilized NCs. This was not due to the “Ostwald ripening” (because it needs aqueous environment). It might be associated with loss of protection of the NCs due to the hydration and swelling of the traces of stabilizer and cryoprotectant [70]. Non-significant (*p* < 0.05) changes in the polydispersity index, surface charges and drug content was found even at stressed condition (higher temperature) at 3 and 6-month. The drug content determination has shown that the OLA was present approximately 97.67% at 6 months even at 40 °C, representing that the freeze-dried OLA-NCs (NCs-2) was chemically stable with no chemical degradation/modification of the drug. The results for drug content analysis indicated that the process of homogenization and probe sonication to prepare the NCs could not disrupt the chemical stability of the drug [71,72,73]. Conclusively, the OLA-NCs (NCs-2) was stable chemically (drug content) as well as physically (size, PDI and ZP) at the storage temperatures. Thus, the developed and freeze-dried OLA-NCs can be stored for 6 months even at room temperature without significant loss of drug content and the physical characteristics of the drug NCs.

### 3.9. MTT Assay

**Figure 8:**
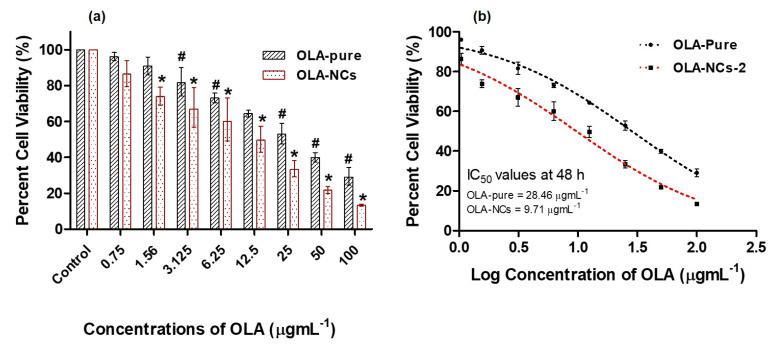
The cell viability assays of OLA-pure and OLA-NCs by considering the 100% cell viability in the control group. Data were presented as mean of three readings with standard deviations (Mean ± SD, *n* = 3). * *p* < 0.05 designates the significant difference between OLA-NCs vs. OLA-pure (except at 0.75 μg·mL^−1^) and the control (untreated cells) while ^#^ *p* < 0.05 designates the significant difference between OLA-pure vs. the control (untreated cells) (**a**). The cytotoxicities for OLA-pure and OLA-NCs with their IC_50_ values (µg·mL^−1^) after 48 h of incubation with MCF-7 cells (**b**).

The cytotoxicity of the pure-OLA and OLA-NCs against the MCF-7 cells (the histograms of cell viability (%) against different concentrations of drug) as well as log drug concentrations versus percent cell viabilities were represented in Figure 8a,b, respectively. The OLA concentrations confirmed a concentration-dependent reduction in the percent cell viability (Figure 8a). The IC_50_ values of pure-OLA and OLA-NCs against the MCF-7 were 28.4 µgmL^−1^ (with *R*^2^ = 0.995) and 9.7 µgmL^−1^ (with *R*^2^ = 0.998), respectively. The results of MTT assay against the MCF-7 cells in the present investigation, indicated a significant increase (*p* < 0.05) in the cytotoxic effect of OLA-NCs as compared to the pure-OLA preparation, except at 0.75 μg·mL^−1^ concentration as compared to the untreated control (*p* > 0.05). The potential increase in the anticancer activity of OLA against MCF-7 cells by OLA-NCs was attributed to the increased solubility and cellular uptake of the drug from NCs form. Therefore, nano-crystallization of OLA (OLA-NCs) could be a potential alternative to the conventional use of pure OLA against the human breast cancer.

### 3.10. P^53^, Caspase-3 and Caspase-9 Assay by ELISA

**Figure 9:**
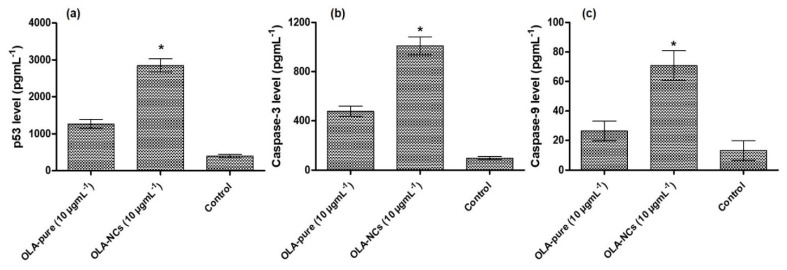
Effects of OLA-pure and OLA-NCs on the activation of p53 level (**a**); Caspase-3 level (**b**) and Caspase-9 level (**c**) in MCF-7 treated cells. The data were represented as mean of three readings with standard deviations (Mean ± SD, *n* = 3). * *p* < 0.05 indicated the significant difference between the activities of OLA-NCs vs. OLA-pure as compared to the untreated (control) cells.

The programmed cell death (apoptosis) specifically disassemble many intra-cellular constituents, while circumventing the injuries and inflammations to the nearby normal cells, which was mainly executed by the activated Caspase-3 and expression of p53 [74]. Therefore, the p53, Caspase-3 and Caspase-9 activities were found to have close linkage with apoptosis of cancer cells as well as chemotherapy based cell death. In the present investigation, a significant increase (*p* < 0.05) in the p53 expression was found in the OLA-pure and OLA-NCs as compared to the untreated group (Figure 9a), which confirmed an efficient apoptotic activity of OLA-NCs > OLA-pure against MCF-7 cells i.e., the p53 protein expressions were 3.2- and 7.2-times higher when the cells were treated with OLA-NCs and OLA-pure, respectively, as compared to the control group.

In addition, significant increase (*p* < 0.05) in the Caspase-3 (Figure 9b) and Caspase-9 (Figure 9c) protein expressions were noted in case of OLA-NCs as well as OLA-pure treated MCF-7 cells as compared to the control (untreated), which further confirm the significant apoptotic activities of OLA-NCs > OLA-pure. When the MCF-7 cells were treated with OLA-pure and OLA-NCs, approximately 4.3-folds and 9.3-folds increased activities of Caspase-3; 2-folds and 5.3-folds increased expression of Caspase-9, respectively, were noted as compared to the untreated (control) cells, which was in agreement with a previous studies [75]. The increased expressions of p53, Caspase3 and Caspase-9 in the cases of OLA-NCs treated group as compared to the other groups, might be associated with high solubility of NCs, which could provide a sufficiently higher concentration of the drug around the treated cells. The sufficiently higher concentrations could saturate and prevent the Pgp-mediated efflux of OLA (a Pgp-substrate) [4,60,76]. Conclusively, an improved effectiveness of OLA from its NCs signifies the possible reason for its successful application to induce the apoptotic cell death in breast cancer, which would be successfully utilized for the treatment of many other cancers including the ovarian and prostate cancers.

### 3.11. In Vivo Pharmacokinetics

The reported reverse phase HPLC-UV method was successfully employed for the quantification of OLA in rat plasma samples [7,20]. The OLA and the IS were eluted at the retention time of 4.55 min and 7.25 min, respectively. The adopted method has shown good linearity in the calibration range 10–2500 ng·mL^−1^. The concentration of OLA was calculated by considering the OLA/IS ratio, using the straight line equation; y = 0.022x − 0.191, *R*^2^ = 0.9993.

**Figure 10:**
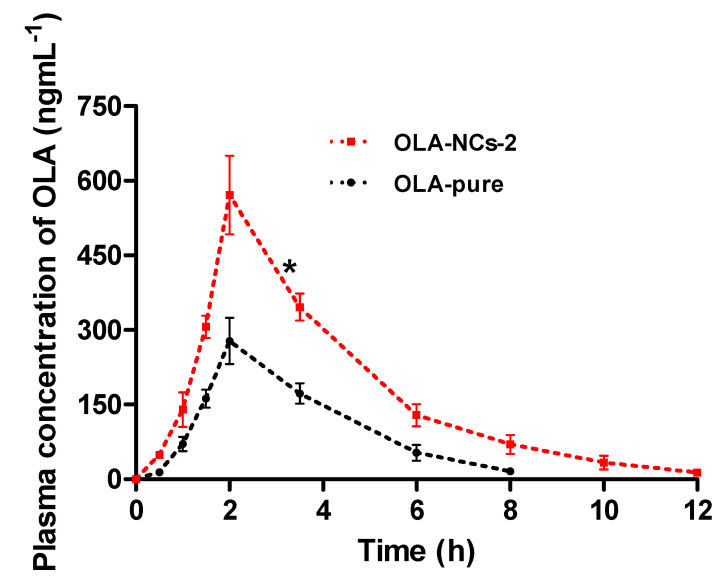
The drug concentration-time profiles of plasma samples oral administration of OLA-pure (conventional AqS) and OLA-NCs-2 in rats. Data are the mean of three samples (three animals per group) with standard error of mean (Mean ± SEM, *n* = 3). * (*p* < 0.005) OLA-NCs-2 vs. conventional AqS of OLA-pure.

The efficiency of OLA-NCs as compared OLA-pure after their oral administration in rats was evaluated in terms of pharmacokinetic profiling of OLA. The equivalent amounts of OLA were suspended in PBS to get the dose of OLA (25 mg·kg^−1^ body weight). The drug concentration in plasma versus time profiles of OLA were represented in Figure 10, and the pharmacokinetic parameters were summarized in Table 5.

After oral administration, the OLA-NCs demonstrated a fast release indicating the fast absorption of OLA and achieved a maximum concentration (C_max_) approximately 571.5 ng·mL^−1^ at Tmax (2 h) as compared to the AqS of OLA-pure (C_max_, 278.08 ng·mL^−1^ at the same Tmax). A significant increase (*p* < 0.005) in the C_max_ was noted from NCs, which was approximately 2.06-folds. Thereafter, the concentration of drug from both the formulation was decreased in log-linear fashion with slightly fast elimination half-lives, which was 1.36 h for OLA-pure as compared to 1.85 h for OLA-NCs.

The statistically significant (*p* < 0.005) differences were found in the other parameters also, except in the MRT_0-∞_, which were almost equal for OLA-AqS and OLA-NCs i.e., 3.37 h and 3.94 h, respectively. Approximately 2.29-, 2–25- and 2.62-folds increased AUC_0–12 h_, AUC_0-∞_ and AUMC_0-∞_, respectively, were calculated for the drug from the NCs as compared to AqS of pure drug. Contrary to the above findings, the clearance rate (CL/F) of OLA was 2.27-times faster from the OLA-pure (6.94 L·h^−1^) as compared to the OLA-NCs (3.05 L·h^−1^). The faster clearance of the drug from AqS of OLA-pure could be the primary reason for its relatively lower bioavailability, which was further confirmed by the fast elimination of the drug, as the concentration was detected only up to 8 h plasma samples and it was remained undetected in 10 h and 12 h samples obtained from OLA-pure treated rats.

Although the elimination half-life t_1/2_ (h) and MRT_0-∞_ for both the formulation were almost equal still, a significant increase in the overall bioavailability of OLA was found from the NCs as compared to its counter formulation after their oral administration in rats. The primary reason behind this achievement was the increased saturation solubility of OLA from the NCs as compared to OLA-pure. The crystal sizes of OLA-NCs in the nano-range was attributed to its increased solubility in around the gastro-intestinal tract. The high solubility of NCs could provide a sufficiently high concentration of OLA in the systemic circulation, and this high concentration could saturate and prevent the Pgp-mediated efflux as OLA is a substrate for P-glycoprotein, which in turn increase the oral bioavailability of OLA [60,76]. This finding is in agreement with a report, where approximately 2-folds increased oral bioavailability of OLA was achieved from the lipid based nano-formulations after its single oral administration in rats [7]. Conclusively, an increased bioavailability of OLA from its NCs suggests a possible reason for its successful use to encourage the apoptotic cell death in cancers including the ovarian and prostate cancers.

## 4. Conclusions

Till date Soluplus^®^ was not used as stabilizer for the nano-crystallization of poorly soluble drugs. The nanocrystals of Olaparib (a PARP-I inhibitor) was successfully developed by homogenization followed by probe sonication techniques without adversely affecting the structural and intrinsic anticancer property of the drug. The OLA-NCs significantly increased (*p* < 0.05) the saturation solubility and in vitro release of OLA in the biological buffers (pH 6.8 > pH 1.2) with the stabilizer, which was also evident from in vitro cell line studies. Notably, the potential cytotoxicity of OLA-NCs on MCF-7 cells was proven by MTT assay for cytotoxicity (cell viability) as well as apoptotic activities through the enhanced expression of p53, Caspase-3 and Caspase-9 by ELISA kits. The OLA-NCs significantly induced pro-apoptotic proteins Caspase-3 and Caspase-9 along with anti-apoptotic protein (p53) as compared to OLA-pure; hence, OLA-NCs was more cytotoxic as compared to OLA-pure. The significant increase in the Cmax, AUC_0–12 h_ and reduced clearance rate further endorsed the improved cytotoxic effect on MCF-7. Therefore, OLA-NCs are promising chemotherapeutic agent against breast cancer. The findings of the present study need further pre-clinical and clinical interpretation.

## Figures and Tables

**Table 1:** Characterization of the OLA-NCs prepared by homogenization followed by probe sonication with two different concentrations of Soluplus^®^. Data were represented as mean of three measurements with standard deviation (Mean ± SD, *n* = 3).

Formulations	Amount of		Physical Characterization (Mean ± SD, *n* = 3)			Drug Content (%)
	OLA (mg)	Soluplus (%, *w*/*v*)	Particle Size (nm)	Polydispersity INDEX	Zeta Potential (mV)	
NCs-1	25 mg	12.5 mg (0.25%)	176.03 ± 8.81	0.202 ± 0.021	+8.59 ± 0.84	94.54 ± 3.95
NCs-2	25 mg	25 mg (0.5%)	92.43 ± 7.02	0.056 ± 0.091	+8.46 ± 1.27	98.67 ± 1.68
OLA-AqS	25 mg	Tween-80 (25 mg) in 10 mL of PBS	402.63 ± 13.52	0.421 ± 0.064	−4.06 ± 0.16	91.65 ± 3.21

NCs = Nanocrystals, OLA = Olaparib.

**Table 2:** Changes in the characteristics of OLA-NCs-2 after freeze-drying. Data are represented as mean of three measurements with standard deviation (Mean ± SD, *n* = 3).

Parameters	Before Freeze-Drying	After Freeze-Drying with Mannitol
Size (nm)	92.43 ± 7.02	103.13 ± 9.41
Polydispersity index	0.056 ± 0.091	0.104 ± 0.061
Zeta Potential (mV)	+8.46 ± 1.27	+8.67 ± 1.25
Drug content (%)	98.67 ± 1.68	98.52 ± 1.86

**Table 3:** Release models applied to check the release kinetics of OLA from the OLA-NCs-2 at two pH conditions.

Release Models	At pH 1.2			At pH 6.8		
	*R*^2^ Values	Slope	RE	*R*^2^ Values	Slope	RE
Zero order (Fraction drug released vs. Time)	0.7497	0.0215	NA	0.8251	0.0349	NA
First order (Log% Drug remaining vs. Time)	0.8035	0.0142	0.0162	0.9592	0.0372	0.0062
Korsmeyer–Peppas (Log Fraction drug released vs. log Time)	0.9541	0.6413	0.2818	0.9764	0.6491	0.2784
Hixon–Crowell (M_o_^1/3^–M_t_^1/3^ vs. Time)	0.7861	0.0094	NA	0.9208	0.0206	NA

RE = Release exponents, NA = Not applicable.

**Table 4:** Stability results of freeze-dried OLA-NCs-2. The data are the mean of three measurements with standard deviation (Mean ± SD, *n* = 3).

Stability of OLA-NCs-2		At Different Time Points (Mean ± SD, *n* = 3)			
Parameters	Initially	At 7th Day	At 1-Month	At 3-Month	At 6-Month
	Mean ± SD	Mean ± SD	Mean ± SD	Mean ± SD	Mean ± SD
**At 4 ± 2 °C**					
Size (nm)	103.13 ± 9.41	103.23 ± 9.43	104.33 ± 9.86	106.60 ± 10.85	108.93 ± 11.03
Polydispersity index	0.104 ± 0.061	0.106 ± 0.062	0.109 ± 0.061	0.131 ± 0.079	0.146 ± 0.084
Zeta potentials (mV)	+8.67 ± 1.25	+8.57 ± 1,25	+8.46 ± 1.26	+8.30 ± 1.15	8.31 ± 1.16
Drug content (%)	98.53 ± 1.86	98.44 ± 0.81	98.34 ± 1.74	98.24 ± 0.72	97.69 ± 1.56
**At 25 ± 1 °C**					
Size (nm)	103.13 ± 9.41	103.5 ± 9.56	106.43 ± 10.31	109.06 ± 9.96	111.53 ± 10.57
Polydispersity index	0.104 ± 0.061	0.108 ± 0.061	0.131 ± 0.074	0.137 ± 0.075	0.163 ± 0.081
Zeta potentials (mV)	+8.67 ± 1.25	+8.63 ± 1.11	+8.53 ± 1.12	+8.33 ± 1.09	+8.17 ± 1.04
Drug content (%)	98.53 ± 1.86	98.04 ± 1.53	97.82 ± 1.37	97.41 ± 1.29	97.01 ± 1.04
**At 40 ± 1 °C**					
Size (nm)	103.13 ± 9.41	105.37 ± 9.71	107.26 ± 10.35	109.43 ± 10.64	112.87 ± 11.28
Polydispersity index	0.104 ± 0.061	0.115 ± 0.064	0.132 ± 0.076	0.139 ± 0.076	0.167 ± 0.082
Zeta potentials (mV)	+8.67 ± 1.25	+8.53 ± 1.11	+8.43 ± 1.12	+8.37 ± 1.27	+8.16 ± 1.21
Drug content (%)	98.53 ± 1.86	98.01 ± 1.51	97.72 ± 1.49	97.68 ± 1.55	97.67 ± 1.58

**Table 5:** Oral pharmacokinetic of OLA-formulations. The data were represented as mean of three readings with standard error of mean (Mean ± SEM, *n* = 3), where * (*p* < 0.005) represents the significant difference between NC_1_ as compared to conventional AqS.

Pharmacokinetic Parameters	For OLA-Pure (Mean ± SD, *n* = 3)	For OLA-NCs (Mean ± SD, *n* = 3)	Enhancement Ratio
t_1/2_ (h)	1.36 ± 0.04	1.87 ± 0.11	1.36
T_max_ (h)	2.00 ± 0.00	2.00 ± 0.00	Same
C_max_ (ng·mL^−1^)	278.08 ± 46.52	571.51 ± 79.01 *	2.06
AUC_0–12 h_ (ng·mL^−1^·h)	882.04 ± 136.74	2022.19 ± 200.67 *	2.29
AUC_0-∞_ (ng·mL^−1^·h)	913.37 ± 137.56	2058.04 ± 197.62 *	2.25
AUMC_0-∞_ (ng·mL^−1^·h^2^)	3081.17 ± 471.32	8093.86 ± 700.68 *	2.62
MRT_0-∞_ (h)	3.37 ± 0.03	3.94 ± 0.04	1.16
Cl/F (L·h^−1^)	6.94 ± 0.96	3.05 ± 0.28	2.27

## Data Availability

The data presented in this study are available on request from the corresponding author.

## Supplementary Materials

The following supporting information can be downloaded at: https://www.mdpi.com/article/10.3390/polym14224827/s1, Figure S1: Release kinetics of OLA from NCs that followed the Korsmeyer-Peppas models at pH 1.2 and 6.8 (a) and (b), respectively, while second best fit-model was first order at pH 1.2 and 6.8 (c) and (d), respectively.
