# Supplementary material for: Nanocrystallization Improves the Solubilization and Cytotoxic Effect of a Poly (ADP-Ribose)-Polymerase-I Inhibitor
Source: Polymers (Basel). 2022 Nov 9;14(22):4827. doi: 10.3390/polym14224827 (PMC9696361; doi:10.3390/polym14224827)
Supplement: Supplementary file 1 [file polymers-14-04827-s001.zip › polymers-1958436-supplementary.pdf]

Supplementary Materials

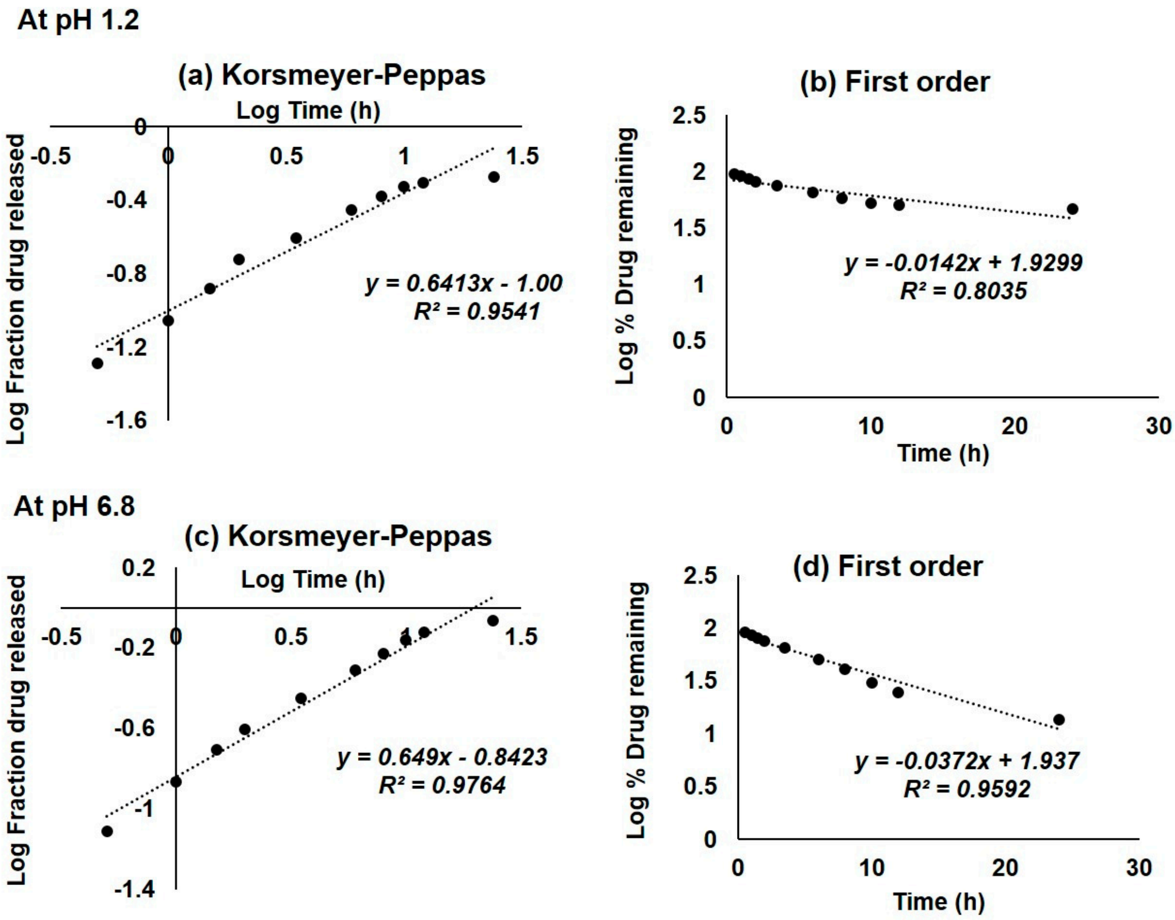

Figure S1. Release kinetics of OLA from NCs that followed the Korsmeyer-Peppas models at pH 1.2 and 6.8 (**a**) and (**b**), respectively, while second best fit-model was first order at pH 1.2 and 6.8 (**c**) and (**d**), respectively.
